# Lightweight MSW-YOLOv8n-Seg: the instance segmentation of maturity on cherry tomato with improved YOLOv8n-Seg

**DOI:** 10.3389/fpls.2025.1731580

**Published:** 2026-01-08

**Authors:** Ronghui Miao, Zhiwei Li

**Affiliations:** 1 College of Agricultural Engineering, Shanxi Agricultural University, Jinzhong, China; 2 College of Information Science and Engineering, Shanxi Agricultural University, Jinzhong, China

**Keywords:** cherry tomato, instance segmentation, maturity, MobileViTv3, MSW-YOLOv8n-Seg, SK attention, WIoU

## Abstract

**Introduction:**

Automatic and accurate segmentation of cherry tomato maturity in natural environment is the foundation for automatic picking. Lacking of significant differences in adjacent maturity and the problem of mutual occlusion between fruits usually affect the picking process. According to the changes in phenotypic characteristics of cherry tomato during its mature period and the Chinese national standard GH/T 1193-2021, a lightweight maturity instance segmentation method of cherry tomato with 5 levels, including green, turning, pink, light red and red was proposed based on improved YOLOv8n-Seg model, named as MobileViTv3-SK-WIoU-YOLOv8n-Seg (MSW-YOLOv8n-Seg).

**Methods:**

In this model, MobileViTv3 was introduced into the original YOLOv8 model as backbone for feature extraction to reduce the parameters of the original model; selective kernel (SK) attention module was added to the neck part to improve the feature expression ability of the model; the complete intersection over union (CIoU) loss function in the original head part was replaced with wise intersection over union (WIoU), which can effectively filter low-quality samples and improve the stability and reliability of the model in complex scenes. The proposed model can better balance the relationship between segmentation speed, accuracy, and model computational complexity.

**Results:**

The experimental results show that the bounding box precision, recall and mean average precision (mAP)@0.5 of the improved model on the test sets were 90.8%, 86.3% and 83.9% respectively, and the model size was 6.0 MB. Compared with YOLOv7-Mask, YOLOv8n-Seg, YOLOv9s-Seg, YOLO11n-Seg, Mask R-CNN (Mask region-based convolutional neural network) and Mask2Former, the bounding box precision increased by 9.6%, 5.2%, 5.7%, 12.3%, 13.3% and 5.0%, the recall increased by 7.8%, 7.4%, 8.8%, 13.1%, 13.9% and 0.1%, and the mAP@0.5 increased by 10.5%, 3.0%, 0.9%, 15.0%, 13.8% and 1.4% respectively. In terms of inference speed, the MSW-YOLOv8n-Seg has the highest inference speed, with FPS of up to 52.9 f·s^-1^ and latency of only 18.2ms, which demonstrates its real-time processing capability.

**Discussion:**

The results show that the improved MSW-YOLOv8n-Seg model is optimal, and it suitable for instance segmentation scenarios with high real-time performance and can provide effective exploration for automated cherry tomato fruit picking.

## 1 Introduction

As an important component of fruit and vegetable picking robot, visual recognition system plays a crucial role in target recognition and positioning, automatic picking, and yield estimation (Wu et al., 2025). Cherry tomatoes have been widely cultivated due to their high nutritional value and unique flavor (Cheng et al., 2024b). However, the characteristics of cherry tomato fruits are dense, small, inconsistent in height, and severely obstructed by branches and leaves, thus making harvesting the most time-consuming and labor-intensive task (Notshweleka et al., 2024). At present, the harvesting of cherry tomato mainly relies on manual labor, which is costly and inefficient, and cannot guarantee optimal picking. The detection and segmentation of fruit maturity can directly determine the transportation and storage ways, also have a significant impact on the price and flavor of fruits. In contrast to object detection, instance segmentation not only identifies and locates each object in an image, but also assigns a specific label to each pixel of each object to distinguish between different instances, and it further distinguishes different instances of the same object based on object detection. Instance segmentation is a combination of object detection and semantic segmentation, which makes semantic localization more refined. Fine-grained segmentation is sutable for precision agriculture management. At the same time, the discrimination problem caused by the similarity between instances also poses certain challenges. Furthermore, we can obtain the pixel-level mask information of each instance through instance segmentation, and combined with the depth information, three-dimensional target localization can be realized, thus providing a basis for subsequent intelligent harvesting. Therefore, constructing an automatic maturity detection and segmentation system with high recognition accuracy is of great significance for determining the distribution of fruits at different maturity stages.

Since 2012, it has been a period of widespread application of machine vision and spectral analysis technologies, during which more researches have applied these techniques to fruit maturity detection, such as mangoes (Wendel et al., 2018), persimmons (Kaizu et al., 2022), bananas (Arman et al., 2023), strawberries (Fan et al., 2024) and other fruits. Shang et al. (2023) investigated nondestructive detection of the quality attributes and maturity of kiwifruits by using hyperspectral imaging technique and chemometric algorithms, which designed partial least square discriminant analysis and simplified k-nearest neighbor models to discriminate the maturity of kiwifruits, with a classification accuracy of 93.3% and 98.3% respectively. Liu and Meng (2024) combined hyperspectral imaging technology with custom convolutional neural network (CNN-S) model to detect the maturity of korla pear, and compared with traditional convolutional neural network (CNN) models, the CNN-S model improved the accuracy of the test set by nearly 10%. However, most of the feature extraction processes in the above literatures were artificial, with limited feature expression and low generalization ability, making it difficult to perform the detection of fruit maturity under natural conditions.

Currently, deep learning technology holds tremendous advantages in the fields of object detection and instance segmentation, and its ability to extract high-dimensional features of targets makes it possible to identify fruits at different maturity stages under complex conditions. Researchers have applied it to the maturity detection and instance segmentation of pineapples (Cuong et al., 2022; Trinh and Nguyen, 2023), strawberries (Cheng et al., 2024a), pistachios (Gkalp and Mazhar, 2024), and cherries (Cossio-Montefinale et al., 2024), brinjals (Tamilarasi and Muthulakshmi, 2025, Tamilarasi et al., 2025) and other agricultural products. Narasimha and Nayal (2020) proposed an automatic maturity classification method based on deep learning, which could minimize the time cost of maturity classification. The experimental results showed that Roboflow algorithm achieved the best validation accuracy results compared with YOLOv7. Blekos et al. (2023) proposed a new sizeable grape dataset called CERTH which was designed explicitly for evaluating deep learning algorithms in grape segmentation and maturity estimation, and the images in the dataset were captured under various illumination conditions and viewing angles, and there were significant occlusions between grape bunches and leaves, making it a valuable resource for the research community. But the focus of the research was the construction of dataset, and there were too many general object detection models used in the experiments, lacking in-depth comparative studies on the performance of the models. Similarly, Zhai et al. (2024b) introduced a novel blueberry ripeness and counting detection methodology that integrated an attention mechanism with a bidirectional feature pyramid network (BiFPN) within the YOLOv5 framework, and the proposed model demonstrated a proficient detection of both the ripeness stages and the quantity of blueberry fruits, providing a foundational application for the development of automated blueberry harvesting techniques in real-world scenarios. Wei et al. (2024) proposed a lightweight tomato maturity detection model GFS-YOLO11 based on improved YOLO11, it can not only accurately identify tomatoes with different maturity, but also effectively distinguish between common tomatoes and cherry tomatoes, which shows strong adaptability to complex field environments. However, it only divided the maturity of tomatoes into three levels (fully mature, semi-mature, and immature), according to the Chinese national standard of GH/T 1193-2021, tomatoes can be further subdivided, and considering the correlation between maturity levels and the storage time and transportation ways, refined maturity classification can make the harvester operations more precise.

As mentioned above, existing researches on fruit maturity detection has made certain progress, there are still problems to be solved. Object detection can only obtain the position and class information of the target, and cannot estimate its picking posture. While, instance segmentation can simultaneously obtain the position, class, and mask information of the target. Based on this, it can further obtain the picking posture, and combine it with the depth information from RGBD camera to guide the operation of the robotic arm, achieving picking sequence planning. Deep learning-based object detection and segmentation models have significant advantages in fruit maturity detection. Among them, with high detection accuracy and speed, and support for high-resolution images, YOLOv8 has been widely used in pedestrian detection, industrial product defect detection and other fields. Therefore, YOLOv8 model was selected as the baseline network, and an improved instance segmentation model for cherry tomato maturity was proposed. The main contributions are as follows:

1. Construction of fine cherry tomato maturity levels. According to the classification of tomato maturity levels in the Chinese national standard GH/T 1193-2021, as well as the analysis of cherry tomato characteristics, we defined the maturity of cherry tomato into 5 levels according to the change in pericarp color, namely green, turning, light red, pink and red. The classification of maturity levels is closely related to processing, storage, and transportation, therefore timely harvesting is of great significance for subsequent agricultural production.
2. Design of lightweight model. Most of the existing fruit maturity detection and segmentation methods based on convolutional neural networks have high detection accuracy, but low detection speed and high model computational complexity; however, methods with low computational complexity and fast detection speed have low detection accuracy. To balance the relationship between detection speed, accuracy, and model computational complexity, the backbone feature extraction network of YOLOv8 was replaced with a lightweight network MobileViTv3, which can significantly reduce the model size and computational complexity.
3. Solution of the difficulty of adjacent maturity identification. Due to the susceptibility of cherry tomato to light, temperature and other environmental factors during their growth, the maturity of fruits in the same inflorescence is inconsistent and there is no significant difference in the characteristics of adjacent maturity. SK attention was introduced into the neck part of YOLOv8n-Seg to enhance the feature expression ability, reduce the influence of low detection accuracy caused by fewer model parameters, and improve the detection accuracy of adjacent maturity and obscured fruits.
4. Optimization of model performance. WIoU loss function was introduced to improve the accuracy and robustness of the model in predicting bounding box.

The remainder of this study is organized as follows. The proposed methods are introduced in detail in Section 2. Section 3 then outlines the experiments performed on cherry tomato images using the proposed method, including ablation studies and comparisons with other modules and models to validate its effectiveness. Section 4 discusses the performance of our method. Conclusions are presented in Section 5.

## 2 Materials and methods

### 2.1 Image acquisition

The experiment was carried out in the tomato research experimental field of shanxi agricultural university in xianggu village, taigu district, jinzhong city, shanxi province in 2024. The main cultivated varieties of cherry tomatoes were red jade, black pearl, camouflage, red pearl, yellow pearl, etc. In this paper, with the highest yield, red pearl was selected as research object, and Oppo Reno5 mobile phone was used to collect images of cherry tomatoes with different maturity under greenhouse from March to May 2024. The collection time included morning, noon and afternoon to ensure the images acquired under different lighting conditions. A total of 321 images of cherry tomatoes with 3468×4624 pixels were obtained. The types of the collected images included single-object, multi-object, fair-light, back-light, with branches and leaves obscured, without branches and leaves obscured, and with fruits obscured, etc. The sample images of cherry tomato are shown in **Supplementary Figure 1**.

### 2.2 Fruit maturity levels

According to the national standard GH/T 1193-2021, tomato maturity is divided into 7 levels based on the color changes of peel and pulp, namely immature, green mature, turning, pre red ripe, mid red ripe, late red ripe, and over ripe stages. Since the focus of this study is to achieve non-destructive detection of cherry tomato maturity in hanging branch state, compared with the determination of maturity by cross-sectional analysis of pulp color, the color changes of pulp cannot be obtained the hanging branch state. Furthermore, compared with tomatoes, cherry tomatoes have a smaller fruit size and shorter growth time, resulting in less difference between adjacent maturity levels. Thus, the 7-levels maturity (**Supplementary Table 1**) was merged into 5-levels based on the actual growth and peel color changes of cherry tomatoes. Among them, the first level of the 7-levels maturity is immature, during which the fruit had not yet fully grown and shaped, making it difficult to accelerate ripening and is unsutable for harvesting, therefore, the first level was deleted; the 1–5 levels of the 5-levels maturity were constructed by the 2–6 levels of 7-levels, which were defined based on the color changes in peel; the 7th level of 7-levels is defined based on the color changes in pulp, then it was deleted. Finally, according to the color changes in peel, the 5-levels maturity was defined: green, turning, light red, pink and red, as shown in **Supplementary Table 2**.

### 2.3 Dataset construction

In order to improve the effectiveness and generalization ability of the model, data augmentation was needed to increase the number of samples, and prevent overfitting problems caused by insufficient training samples (Navarro et al., 2018). Gaussian blur, horizontal flipping, vertical flipping, non-proportional scaling, random translation, perspective transformation, and random cropping were used to expand the dataset, and parts of the data augmentation samples are shown in **Supplementary Figure 2**.

The cherry tomato dataset in this study was a YOLO series format. Therefore, Labelme 5.0.1 software was used to annotate different maturity levels of cherry tomatoes in txt format. Based on the demand for robot picking, whether fruits are picked or not is usually determined by their recognizability, accessibility, and operable space, and the obstruction of fruits and branches directly affects all these factors (Rajendran et al., 2024). In actual harvesting, the occlusion situation is usually divided into three levels: slight occlusion (more than 80% of the fruit area is visible and considered to be harvestable ), moderate occlusion (about 20%-50% of the fruit area is occluded and requires advanced algorithms to predict the complete contour of the fruit and complex path planning algorithms to remove the occluded leaves), and severe occlusion (more than 50% of the fruit area is occluded and considered to be abandoned for harvesting) (Wang et al., 2019). Therefore, the annotation rule are as follows: (1) Annotate fruits with slight and moderate occlusion; (2) Fruits with severe occlusion will not be labeled; (3) Green cherry tomatoes that have not yet grown up would not be labeled. **Supplementary Table 3** shows the annotation results of cherry tomatoes in different maturities. To ensure consistency and reliability of maturity labeling, three horticultural experts were assigned to annotate the dataset separately. When there were inconsistencies in the labeling results, most of the voting results were taken as the final labels.

### 2.4 Model construction for instance segmentation of cherry tomato maturity

Cherry tomato is easily affected by light and various environmental factors during their growth, and there are differences in maturity of the same inflorescence. Most of the images in this study contain multiple maturity levels, and the differences in adjacent maturity are not significant. At the same time, with branch and leaf occlusion, fruit occlusion, it is difficult to accurately detect the maturity of cherry tomato. Although the existing deep learning based convolutional neural network models have high detection accuracy, the computational complexity is high and the detection speed is slow. Due to its fast inference speed and high detection accuracy, YOLOv8 has been widely used in fields such as safety monitoring, autonomous driving, smart homes, and industrial automation. Therefore, YOLOv8 model was selected as the baseline network, an improved cherry tomato maturity instance segmentation model was proposed.

#### 2.4.1 Structure of the YOLOv8-Seg network

Benefiting from new backbone network structure, anchor free detection head, and new loss function (Jo et al., 2024), YOLOv8 model can achieve tasks such as object detection, instance segmentation, and key points detection. **Supplementary Figure 3** illustrates the network structure and details of YOLOv8-Seg, and the structure of YOLOv8 mainly consists of three parts: backbone, neck, and head.

1. Backbone: responsible for feature extraction. A series of convolutional and deconvolution layers are adopted to realize deep feature extraction. The introduction of residual connections and bottleneck structures can improve the performance of the network (Zhai et al., 2024a). Compared with YOLOv5, C2f module is introduced to replace the original C3 module, which can achieve light weighting with fewer parameters. CSP-DarkNet (Cross stage partial darknet) combines the advantages of cross stage local networks and DarkNet. Moreover, the depth wise separable convolution and dilated convolution techniques can further enhance feature extraction capabilities.
2. Neck: located between the backbone and head parts, responsible for feature fusion enhancement. The spatial pyramid pooling fusion (SPPF) can fuse features maps from different stages of backbone to enhance feature representation capabilities (Dong et al., 2024). The path aggregation network (PAN) structure achieves deep fusion between shallow and deep features by constructing bidirectional paths from bottom to top and from top to bottom, improving the accuracy and stability of object detection and segmentation.
3. Head: responsible for producing the final detect results. This part adopts simple convolution, upsampling layers, and the feature maps of the neck part to achieve object detection and segmentation.

#### 2.4.2 Structure of the MSW-YOLOv8n-Seg network

This study proposed an instance segmentation method based on YOLOv8n-seg, named MSW-YOLOv8n-seg. MobileViT is a lightweight visual model based on ViT (Vision transformer) architecture, which is designed for mobile devices and embedded systems, and it can reduce the size and computational requirements of the model while maintaining high performance (Alaca, 2025). Then MobileViTv3 was adopted into the backbone of YOLOv8n-Seg to balance the detection speed, accuracy, and model computational complexity. By dynamically adjusting the size of the convolution kernel, SK attention mechanism can improve the model’s ability to capture multi-scale features (Mun et al., 2023). So, we introduced it into the neck part of YOLOv8n-Seg to effectively capture multi-scale features of cherry tomato at different maturity levels. It can effectively solve the problems of small objects, difficulty in detecting adjacent maturity in complex scenes, thereby improving the accuracy and robustness of segmentation. Faced with complex scenes such as branch and leaf occlusion, fruit occlusion, and different lighting conditions, the bounding box loss function of CIoU is susceptible to affected by noisy data or difficult samples (Du et al., 2021). However, WIoU introduces dynamic non monotonic frequency modulation, which can dynamically adjust weights based on differences in sample quality, thus, effectively alleviating the interference of noisy data (Hu et al., 2024b). We replaced the original bounding box loss function CIoU with WIoU at the head part. The improved MSW-YOLOv8n-Seg model is shown as **Supplementary Figure 4**.

#### 2.4.3 Enhanced backbone with MobileViTv3 module

The purpose of lightweighting is to improve the speed and efficiency of the model while ensuring accuracy, and to deploy the model on edge devices. Lightweight models typically include CNN-based, Transformer-based, and CNN-Transformer-based hybrid architecture models. Although the CNN-based lightweight model is already quite mature and efficient on mobile devices, its receptive field is limited and may become a performance bottleneck. Transformer architecture has stronger global modeling capabilities, which are crucial in handling complex scenes or long-distance dependency relationships. However, its computational complexity is usually high, and it lacks the inherent inductive bias of CNN, making it difficult to train in limited data and requiring high computational resources, so it unsutable for edge deployment scenarios (Zhu et al., 2024). The hybrid architecture combines the efficiency of CNN with the global representation capability of Transformer, achieving a good balance between accuracy, efficiency, and resource consumption. As a typical hybrid architecture, MobileViT can not only significantly reduce the number of parameters and computational complexity of the model, but also can better understand the global features in the image, thereby improving the accuracy and stability of classification, and the performance on unknown datasets is also better. Furthermore, MobileViTv3 introduces a bottleneck structure and simpler activation functions to meet the requirements of computational efficiency and hardware friendliness, making it a “simple and effective” architecture. Based on the above criteria, MobileViTv3 was selected as the backbone network to achieve lightweighting.

The network of MobileViT is mainly composed of ordinary convolutional layer, MV2 module of MobileNetV2, MobileViT module and etc, and it is refined into 5 layers (Sun et al., 2024), as shown in **Supplementary Figure 5** (where the blocks that perform downsampling are marked as ↓2). The input image is first convolved through a standard 3x3 convolutional layer, followed by continuous downsampling and pooling to extract features using multiple MV2 and MobileViT blocks, then connected to a 1x1 convolutional layer. Finally, the classification results are obtained through a full connection layer.

The MV2 module in MobileViTv3 follows the one in MobileNetV2, which adopts a depthwise separable convolution, inverse residual, and linear bottleneck structure. It can effectively extract the local features while reducing the number of model parameters. Its structure is shown in **Supplementary Figure 6**.

MobileViTv3 block is a new version of the MobileViT series models. MobileViTv3 block consists of three sub blocks, namely the local representation block, the global representation block, and the fusion block (Xia et al., 2024), the network structure of MobileViTv3 block is shown in **Supplementary Figure 7**. In which, C, H, and W represent the dimension, height, and width of the input feature map; DW represents depthwise separable convolution.

1. Local representation block. First, a 3 × 3 depthwise convolution operation is used to obtain a local feature map. Then, a 1 × 1 convolution is conducted to reduce the dimensionality of the feature map. Finally, the reduced dimensional feature map will be input into the global representation and the fusion blocks.
2. Global representation block. This module utilizes the global expression capability of Transformer to model the full pixel relationship of the feature map, thus a feature map with global feature expressions can be obtained, and then it is input into the feature fusion block.
3. Fusion block. This block concatenates and fuses the output feature maps of the local representation block and the global representation block, and then adds them to the original feature map to obtain a final map with rich expressions.

In the backbone of this paper, the DarknetBottleneck module in the original C2f was replaced with the MobileViTv3 module, which was called C2f_MobileViTv3 in **Supplementary Figure 4**. And all the original C2f modules were replaced with C2f_MobileViTv3 modules (layers 2, 4, 6 and 8, with channel widths of 128, 256, 512, and 1024 respectively).

#### 2.4.4 Enhanced neck with SK attention module

In the field of deep learning, attention mechanism has become one of the important means to improve model performance (Zhang et al., 2025b). SK attention mechanism captures feature information of different scales through multi branch convolution kernels, and then fuses the weights of different scales through softmax function, allowing each neuron to dynamically adjust its receptive field according to the input (Hu et al., 2024a). It can effectively improve the model performance in image classification, object detection and other tasks (Yu et al., 2025). The network structure of SK attention module is shown in **Supplementary Figure 8**. It mainly consists of three parts: split, fuse, and select.

(1) Split: The original image is convolved by convolution kernels in different scales (such as 3×3 and 5×5) to generate multi-scale feature map, that is, U_1_ and U_2_. The input feature map of X is H×W×C (height, width and channels).

(2) Fuse: The U_1_ and U_2_ feature maps are added, and all the branch information are fused, then the global information S is obtained by global average pooling, finally, the vector Z is obtained by full connection. The calculation formulas are shown in Equations 1 and Equations 2.

(1) $$S_{c}=F_{gp}(U_{c})=\frac{1}{H\cdot W}\sum_{i=1}^{H}\sum_{j=1}^{W}U_{c}(i,j)$$

(2) $$Z=F_{fc}(S)=\delta [B(W_{s})]$$

In which, $U_{c}(i,j)$ is the pixel value of position (i,j) in the feature map $U_{c}$; $F_{gp}$ is global average pooling function; $S_{c}$ is the result of global average pooling; B is batch standardization; δ is Relu function; $F_{fc}$ is fully connected layer function; $W_{s}$ represents a fully connected layer weight matrix, it performs a linear transformation on the input S.

(3) Select: a_c_ and b_c_ are obtained by performing Softmax operation on vector Z, then multiply a_c_ and b_c_ with the original feature U_1_ and U_2_ respectively, we can get Y_1_ and Y_2_. Finally, Y is obtained by feature accumulation. The calculation formulas are shown in Equations 3–5.

(3) $$a_{c}=\frac{e^{A_{c}Z}}{e^{A_{c}Z}+e^{B_{c}Z}}$$

(4) $$b_{c}=\frac{e^{B_{c}Z}}{e^{B_{c}Z}+e^{B_{c}Z}}$$

(5) $$Y_{c}=a_{c}U_{1_{c}}+b_{c}U_{2_{c}}$$

In which, $A,B\in R^{d\times c}$; the feature dimension d is determined by max(c/r,L), where r is the deceleration rate, L is the minimum value of d; $A_{c}\in R^{1\times d}$, represents the c-th line of A; $a_{c}$ is the c-th element in a; $B_{c}$ and $b_{c}$ are the same as above.

Through experimental verification, three SK attention module were added after C2f in the 12, 16 and 20 layers of the neck part, namely layers 13, 17 and 21. The insertion point for attention mechanisms is usually after the C2f module, especially in the P3 and P4 layers. The hyperparameters of SK attention mechanism: the reduction ratio is 16; the number of branches is 2; the minimum channel number L is 32; the convolution kernel size are 3×3 and 5×5.

#### 2.4.5 Enhanced head with WIoU loss function

In the instance segmentation task of YOLOv8-Seg, the loss usually consists of two parts: object detection and segmentation loss (seg_loss). Segmentation loss represents the deviation between the predicted segmentation area and the actual one. Object detection loss includes bounding box loss (box_loss), classification loss (cls_loss), and distribution focal loss (dfl_loss). Bounding box loss represents the deviation between the predicted bounding box and the actual one, and the default loss function is CIoU (Complete intersection over union). Classification loss represents the deviation between the predicted category and the true one. Distribution focal loss is used to optimize the predicted distribution of bounding boxes.

The training data inevitably contains low-quality data, which reduces the generalization performance of the model. The traditional CIoU loss function treats all samples equally during optimization, but in practical detection, the localization error of small targets has a greater impact on IoU and is easily dominated by large targets for loss (Liu et al., 2023). Difficult samples with different maturity and occlusion require higher attention to improve the robustness of the model. WIoU leverages a dynamic non-monotonic focusing mechanism to evaluate bounding box quality, a two-layer attention mechanism to address slowed convergence, and a gradient gain to reduce the effect of detrimental gradients. It can enhance the model’s generalization ability while ensuring high-quality bounding box (Cai et al., 2024). The calculation formulas of WIoU are shown in Equations 6–8.

(6) $$L_{IoU}=1-T_{IoU}$$

(7) $$R_{WIoU}=\exp[\frac{{\left(x_{p}-x_{g}\right)}^{2}+{\left(y_{p}-y_{g}\right)}^{2}}{(W_{g}^{2}+H_{g}^{3})*}]$$

(8) $$L_{WIoU}=R_{WIoU}L_{IoU}$$

In which, $T_{IoU}$ is the intersection over union ratio between the predicted box and the real box; $L_{IoU}$ is the basic loss; $x_{p}$, $y_{p}$ and $x_{g}$, $y_{g}$ are the center point coordinates of the predicted box and the real box respectively; W, H are the width and height of the real box; In order to significantly enlarge the ordinary quality anchor box of $L_{IoU}$, $R_{WIoU}$ is introduced; To prevent $R_{WIoU}$ from generating gradients that hinder convergence, $W_{g}$ and $H_{g}$ are separated from the calculation graph (* indicates this operation).

### 2.5 Experimental environment and parameter settings

The experiments in this study were conducted using an NVIDIA GeForce RTX 3080 GPU paired with 12^th^ Gen Intel(R) Core(TM) i7-12700F@2100MHz processor. The operating system is Windows 11 and its GPU driver is CUDA 11.4, with 12 GB of GPU memory. The software is developed using Python 3.10.4 and PyTorch 2.0.1.

During the training process, the batch size is set to 16, the number of training epochs is 300, the learning rate is 0.001. The optimization strategy is the adaptive moment estimation (Adam), β1 is 0.937, β2 is 0.999, and weight decay is 0.0005. Batch size represents the number of training set images input in each batch; epoch represents the number of iterations during the training process; learning rate is a parameter that controls the size of the gradient descent step in each iteration; momentum can accelerate the update of model parameters. The number of mask prototypes in segmentation head is 32, the stride is 4, the upsampling method is nearest neighbor interpolation. The training input resolution policy is fixed. The mosaic and mixup probabilities of augmentations are 1.0. The NMS (Non-maximum suppression) threshold is 0.7.

### 2.6 Model evaluation indicators

In order to evaluate the performance of detection and segmentation, mAP, precision (P) and recall (R) were selected as evaluation indicators. TP (True positives) is the number of samples correctly identified as positive; TN (True negatives) is the number of samples correctly identified as negative samples; FP (False positives) is the number of samples incorrectly identified as positive; FN (False negatives) is the number of samples incorrectly identified as negative samples.

Among them, mAP refers to the average precision at different recall rates. P represents the proportion of samples predicted as positive cases that are actually positive ones. R represents the proportion of samples correctly identified as positive cases by the model among all actual positive cases (Ma et al., 2025). The calculation formulas are shown in Equations 9 to Equations 10.

(9) $$P=\frac{TP}{TP+FP}$$

(10) $$R=\frac{TP}{TP+FN}$$

The average precision (AP) for a single category was calculated by ranking the model predictions based on their confidence scores, and determining the area under the precision-recall (PR) curve. The calculation formulas are shown in Equations 11.

11) $$AP=\int_{0}^{1}P(R)d(R)$$

Mean average precision (mAP) is the mean of AP which was calculated across all categories, then offering a comprehensive evaluation of the model’s performance. The calculation formulas are shown in Equations 12.

(12) $$mAP=\frac{1}{n}\sum_{k=1}^{k=n}AP_{k}$$

In which n is the number of categories.

## 3 Results

### 3.1 Quantitative and qualitative evaluation of MSW-YOLOv8n-Seg model

**Supplementary Figure 9** shows the loss curves of MSW-YOLOv8-Seg model on the training sets. The loss functions of the proposed method include four types of loss: bounding box loss (box_loss), segmentation loss (seg_loss), classification loss (cls_loss), and distribution focal loss (dfl_loss). As shown in **Supplementary Figure 9**, with the increase of training epochs, the loss value rises first and then continuously decreased, finally tended to stabilize, which indicates that the performance of the model is gradually improving in the classification and prediction tasks.

To evaluate the generalization ability of and reduce the risk of overfitting, this paper conducted cross-validation experiments on the MSW-YOLOv8n-Seg model. Five independent training sets (seeds data) were randomly generated while keeping the ratio of the original training sets to the validation sets constant, and the statistical results were evaluated on the corresponding validation sets. The specific results of the cross-validation experiments are presented in **Supplementary Table 4**, and the statistical results of the evaluation indicators are reported as mean ± standard deviation. In object detection, “box” refers to the matching degree between the predicted bounding box and the actual bounding box, and it is used to evaluate the localization ability of the model. In segmentation task, “mask” refers to the matching degree between the predicted mask area and the actual target area, focusing on the matching of details inside the target. It can be seen that the detection and segmentation performance are consistent in precision, recall, and mAP@0.5, with small standard deviations (≤1.0), indicating that the model performs stably in five different random seed experiments. The precision and recall of box and mask are both high, with precision of 90.3% and recall of 86.9% respectively. Under strict criteria mAP@0.5:0.95, the segmentation performance is lower than the detection performance, with 79.2% and 78.3% respectively, box is 0.9 percentage points higher than mask. The experimental results show that the model performs robustly in detection and segmentation tasks and can meet high-precision requirements.

**Supplementary Table 5** shows the segmentation results of MSW-YOLOv8n-Seg model on the test sets. The total precision, recall, mAP@0.5 of bounding box at different maturity levels can achieve 90.8%, 86.3% and 83.9%, and these represent an excellent outcome. The bounding box values of precision, recall and mAP@0.5 at green maturity are 98.6%, 92.2% and 90.3%, which are the highest, because during this level the surface color of cherry tomato is relatively stable and there is no color change. The values of precision, recall and mAP@0.5 at light red maturity are 77.0%, 67.2% and 61.3%, which are the worst, as the early level is turning, and the later level is pink, with rapid epidermis color changes and short cycles making detection difficult. The detection results of mask are basically the same as that of bounding box. The experimental results show that this algorithm can achieve segmentation at different maturity levels well, providing a basis for subsequent intelligent harvesting. **Supplementary Figure 10** shows the confusion matrix and PR curve of MSW-YOLOv8n-Seg on the test sets, in which, 10(a) is the confusion matrix, and 10(b) is the PR curve of bounding box. In **Supplementary Figure 10a**, the horizontal and vertical axis represents the true and the predicted value respectively, and the diagonal elements represent the number of correct predictions. It can be seen that, pink performs the best; the green category performed well, with 7 Green categories miss classified as background; light red performs the worst, with misjudgments and missed detections; there are also misjudgments and missed detections in the red category. The PR curve can verify the overall performance of the model. Consistent with the confusion matrix results, pink and green have the highest AP values, and the curve is closest to the upper right corner, indicating that the model can recognize these two categories with high credibility; red and turning are both higher than the overall value, indicating a stable and reliable performance; light red performs the worst. The PR curve indicates that the overall performance of the model is good, but there is a certain degree of category imbalance.

To better evaluate the detection and segmentation performance of the improved MSW-YOLOv8n-Seg model in complex scenarios, we further selected four sub-datasets of different scenarios: fair-light, back-light, branches and leaves obscured, and multi-object with fruits obscured. The experimental results are shown in **Supplementary Table 6**. According to **Supplementary Table 6**, the back-light scenario performs the best, with a box mAP@0.5 of 92.3%. The branches and leaves obscured performs the worst, with a box mAP@0.5 of 82.7%, and the mAP@0.5:0.95 difference between mask and box can reach 4.3%. The box detection results are generally better than the mask segmentation results. The experimental results show that the model performs well under good lighting conditions, but its performance decreases in the case of branch and leaf occlusion.

### 3.2 Comparison of different instance segmentation models

To compare the segmentation performance of MSW-YOLOv8n-Seg to other models, we selected state-of-art YOLO series and non-YOLO models for quantitative and qualitative comparisons in terms of evaluation metrics and visual effects. YOLO series models included YOLOv7-Mask (Chaithanya and Devi, 2025), YOLOv8n-Seg, YOLOv9s-Seg (Huang et al., 2024), and YOLO11n-Seg (Zhang and Yang, 2021; Zhang et al., 2025a), and these models are well-suited for embedded and edge devices, as they strike a balance between detection accuracy and computational constraints. Non-YOLO models included Mask R-CNN (Bi et al., 2022) and Mask2Former (Guo et al., 2024). In which, the backbone network of Mask R-CNN and Mask2Former was ResNet-50.

The quantitative results are summarized in **Supplementary Table 7**. The segmentation result of and Mask R-CNN and YOLOv11-Seg are relatively poor, and YOLOv7-Mask is also not ideal. The YOLOv9s-Seg model achieved a performance of 85.1% precision, 77.5% recall, 83.0% mAP@0.5 on bounding box, and the segmentation results are satisfactory, however, the GFLOPs is 71.5, which is the highest, the model size is also high with a value of 47.9 MB, indicating that the computational complexity of the model is relatively high. The result of YOLOv8n-Seg is not significantly different from YOLOv9s-Seg, but its model size is 9.8MB, which is significantly better than YOLOv9s-Seg. The result of MSW-YOLOv8n-Seg is optimal, and its model size is also optimal. Compared with YOLOv7-Mask, YOLOv8n-Seg, YOLOv9s-Seg, YOLO11n-Seg, Mask R-CNN and Mask2Former, the value of box precision increased by 9.6%, 5.2%, 5.7%, 12.3%, 13.3% and 5.0%, the recall increased by 7.8%, 7.4%, 8.8%, 13.1%, 13.9% and 0.1%, and the mAP@0.5 increased by 10.5%, 3.0%, 0.9%, 15.0%, 13.8% and 1.4% respectively. In terms of inference speed, the MSW-YOLOv8n-Seg has the highest inference speed, with FPS of up to 52.9 f·s^-1^ and latency of only 18.2ms, which demonstrates its real-time processing capability. Compared with other models, the MSW-YOLOv8n Seg model achieves a good balance in accuracy, speed, and model lightweighting, making it sutable for instance segmentation scenarios with high real-time performance.

**Supplementary Figure 11** directly shows the comprehensive comparison performance of different models. The evaluation indicators include bounding box precision, recall, mAP@0.5, FPS, GFLOPs and model size, and they have been normalized. Although models, such as YOLOv8n-Mask and YOLOv9s-Seg, also achieved excellent results in precision and recall, the MSW-YOLOv8n-Seg model is superior in the terms of comprehensive performance. Especially in terms of model size, the proposed method is more prominent, which is crucial for model deployment and real-time performance in practical applications.

**Supplementary Figure 12** shows the segmentation comparison with different models, and the image covers different lighting conditions and occlusion situations. Each cherry tomato instance is segmented with different color, and the object detection box is labeled with the category and confidence value. It can be seen that YOLOv8n-Seg and YOLOv9s-Seg can effectively segment the cherry tomato instances at different maturity levels with high overall recognition rates, there are still missed detections. YOLO11n-Mask model can segment each instance, but the overall detection confidence is low, and there is a certain degree of duplicate detections. Mask R-CNN has serious missed detections for green targets and far small targets. Mask2Former also has some missed detections for far small targets. The improved MSW-YOLOv8n-Seg is capable of segmenting small and distant targets with high detection confidence, resulting in an optimal performance. These comparative experiments demonstrate the superiority of the improved model in terms of accuracy, efficiency, and memory usage in cherry tomato maturity segmentation.

### 3.3 Comparison of different YOLOv8-Seg scales

This study compared and analyzed the performance results of the improved method with different YOLOv8-Seg scales models. **Supplementary Table 8** shows the comparison results of different YOLOv8-Seg size models. It can be seen that the lightweight MSW-YOLOv8-Seg model performs the best in all evaluation metrics for image segmentation. The detection precision of bounding box is 90.8%, which is 5.2%, 11.3%, 6.5%, 12.4%, and 31.1% higher than YOLOv8n-Seg, YOLOv8s-Seg, YOLOv8m-Seg, YOLOv8l-Seg, and YOLOv8x-Seg, respectively. The detection recall of bounding box is 86.3%, which is 7.4%, 4.8%, 5.7%, 9.7%, and 8.2% higher than YOLOv8n-Seg, YOLOv8s-Seg, YOLOv8m-Seg, YOLOv8l-Seg, and YOLOv8x-Seg, respectively. The values of mAP@0.5 and mAP@0.5:0.95 of the improved model are also the highest, indicating that the model can recognize small targets of cherry tomato at different maturity levels. Similarly, the precision, recall, mAP@0.5 and mAP@0.5:0.95 of mask are also higher than other models. Due to the replacement of the C2f module in the backbone network with a lightweight MobileViTv3 module, and the addition of attention mechanism in the neck part, the model in this paper can improve the detection performance while reducing the number of model size. The model size of the improved model is 6.0MB, which is lower than other models. Usually, as the model scales increased, the model size also increased. Compare with other models, YOLOv8n-Seg has the lowest model size, making it well-suited for future embedded and edge devices. Furthermore, it is compatible with small and medium-sized data and achieves a balance between detection accuracy and resource demands. YOLOv8m-Seg, YOLOv8l-Seg, and YOLOv8x-Seg are more suitable for tasks in complex scenes. The overall detection accuracy of YOLOv8x-Seg is relatively low, with the highest model size, which indicates that larger model scale invariably leads to better performance. Considering accuracy, hardware resources, and subsequent tasks, YOLOv8n-Seg is more suitable for further research on cherry tomato.

**Supplementary Figure 13** shows the convergence of the loss curves for different YOLOv8-Seg size models on the training sets. It can be observed that YOLOv8x-Seg has the slowest convergence, and YOLOv8l-Seg is also relatively slow. In contrast, the convergence speeds of YOLOv8n-Seg YOLOv8s-Seg, and YOLOv8m-Seg are comparable to each other. The convergence speed of the improved model is the fastest. The convergence trends of different types of loss are roughly the same.

### 3.4 Comparative experiments of different modules in backbone

To evaluate the effectiveness of the MobileViTv3 module in the backbone network, the DarknetBottleneck module in the original C2f of YOLOv8n-Seg was replaced with the MobileViTv3 module. This paper compared and analyzed the impact of Biformer (Zhu et al., 2023), FocalNeXt (Bai et al., 2023), ConvNeXtv2 (Pokhrel et al., 2023), GhostNetv2 (Tang et al., 2022), and MobileViTv3 modules on model performance. The experimental results are shown in **Supplementary Table 9**. It can be seen that compared with the baseline network (YOLOv8n-Seg), the introduction of Biformer, FocalNeXt, ConvNeXtv2, GhostNetv2, and MobileViTv3 modules can appropriately reduce the model size and achieve lightweighting. GhostNetv2 and MobileViTv3 achieve the most significant model size reduction, each with a size of 6.0 MB. In contrast, the Biformer, FocalNeXt, and ConvNeXtv2 modules exhibit substantial declines across most evaluation metrics. Furthermore, the accuracy of the GhostNetv2 module remains comparable to that of the baseline. The MobileViTv3 module can improve model detection accuracy while reducing the model size. Compared with YOLOv8n-Seg, its bounding box precision, recall, mAP@0.5, mAP@0.5:0.95, and mask precision, recall, mAP@0.5, mAP@0.5:0.95 increased by 1.6%, 2.0%, 2.1%, 1.7%, and 1.6%, 2.0%, 1.9%, 2.9%, respectively.

### 3.5 Comparative experiments of different attention mechanisms in neck

In order to verify the effectiveness of SK attention mechanism, this study added attention mechanisms into the neck part and replaced the SK attention mechanism with CBAM (Convolutional block attention module) (Wang et al., 2022), EMA (Efficient multi-scale attention) (Zhang et al., 2024), SA (Spatial attention) (Zhang and Yang, 2021; Zhang et al., 2025), and SimAM (Similarity aware attention module) (Mahaadevan et al., 2023) at the same position. Six comparative experiments were conducted based on MobileViTv3-YOLOv8n-Seg (M-YOLOv8n-Seg). The experimental results are shown in **Supplementary Table 10**. It can be seen that the CBAM, EMA, SA, and SimAM modules reduce the detection precision of bounding box in the M-YOLOv8n-Seg model, while the recall rates of CBAM and SA are slightly improved, and the recall rates of EMA and SimAM are both reduced. The mAP@0.5 of the CBAM, EMA, SA and SimAM modules is not much different from that of M-YOLOv8n-Seg. In contrast, the SK attention mechanism outperforms the others, the bounding box precision, recall, and mAP@0.5 are 89.8%, 84.1%, and 85.9%, respectively, which are 2.6%, 3.2%, and 2.9% higher than M-YOLOv8n-Seg. In terms of inference speed, except for the high latency of SimAM with 28.8 ms, the values of other attention mechanisms are not significantly different. The experimental results indicate that the M-YOLOv8n Seg model enhanced with SK module provides the best performance and delay balance.

### 3.6 Comparative experiments using different loss functions in head

In order to verify the impact of regression box loss function on model performance, this paper conducted six comparative experiments based on the MobileViTv3-SK-YOLOv8n-Seg-CIoU (MS-YOLOv8n-Seg) model, including CIoU, DIoU (Distance intersection over union) (Zheng et al., 2020), EIoU (Enhanced intersection over union) (Qi et al., 2023), GIoU (Generalized intersection over union) (Qian et al., 2023), SIoU (Structured intersection over union) (Do et al., 2024), and WIoU. CIoU is the default regression box loss function in YOLOv8n-Seg. **Supplementary Figure 14** shows the loss and mAP@0.5 curves corresponding to different loss functions. From **Supplementary Figure 14**, it can be seen that all loss functions have a clear convergence trend. When the training epochs are in the range of 0-200, the convergence speed is fast. When the training epochs are in the range of 200-300, the loss values of all models tend to stabilize. The loss value of WIoU is optimal, and its mAP@0.5 is also optimal.

**Supplementary Table 11** presents the quantitative experimental results of different loss functions. It can be seen that DIoU, EIoU, and GIoU show a decrease in some indicators. SIoU and WIoU have a certain degree of accuracy improvement, with SIoU’s bounding box precision, recall, mAP@0.5, and mAP@0.5:0.95 being 89.9%, 85.2%, 84.9%, and 78.8%, respectively. Compared to the MS-YOLOv8n-Seg model, the bounding box precision, recall, and mAP@0.5:0.95 of SIoU have improved by 0.1%, 1.1%, and 0.1%, respectively, and the WIoU improved by 1.0%, 2.2%, and 1.1%, respectively. These results demonstrate that WIoU outperforms CIoU, DIoU, EIoU, GIoU and SIoU in cherry tomato maturity segmentation.

### 3.7 Ablation experiments

Based on the comparative experimental results in **Supplementary Table 9**-**11**, it is necessary to verify the effectiveness of each improved module in cherry tomato maturity segmentation. To further verify the impact of different modules on the results, this paper conducted eight ablation experiments based on YOLOv8n-Seg, MobileViTv3, SK, and WIoU, and the design of the ablation experiments is shown in **Supplementary Table 12**.

When replacing the C2f module in the backbone network with MobileViTv3, the bounding box precision, recall, mAP@0.5, mAP@0.5:0.95, mask precision, recall, mAP@0.5 and mAP@0.5:0.95 increased by 1.6%, 2.0%, 2.1%, 1.7%, 1.6%, 2.0%, 1.9%, and 2.9%, respectively. Adding SK attention mechanism to the neck can improve the performance of the model appropriately, with improvements of 3.2%, 0.6%, and 1.3% in bounding box recall, mAP@0.5, and mAP@0.5:0.95, respectively, but a decrease of 0.4% in precision. Replacing CIoU with WIoU (No.4), and using SK and WIoU (No.6) have little effect on model performance, and there is no significant difference in each indicator compared to YOLOv8n-Seg. Comparative analysis of experiments No.1, 2, 5, 7 and 8 shows that the addition of MobileViTv3 module can significantly improve model performance. While the performance improvement effect of No.3, 4, and 6 models are not significant under the original C2f module. By adding both MobileViTv3 and SK modules, the bounding box precision, recall, mAP@0.5, and mAP@0.5:0.95 improved by 4.2%, 5.2%, 5.0%, and 4.3%, respectively. By adding both MobileViTv3 and WIoU modules, the bounding box precision, recall, mAP@0.5, and mAP@0.5:0.95 improved by 4.1%, 6.3%, 4.0%, and 5.3%, respectively. By adding MobileViTv3, SK, and WIoU modules simultaneously, the bounding box precision, recall, mAP@0.5, and mAP@0.5:0.95 improved by 5.2%, 7.4%, 3.0%, and 5.4%, respectively. The experimental results indicate that the addition of the improved modules is of great significance in enhancing the performance of the original model.

## 4 Discussion

In this study, a lightweight cherry tomato maturity instance segmentation model MSW-YOLOv8n-Seg was proposed, and satisfactory results were achieved on the self-built dataset. Through ablation experiments, we verified the effectiveness of MobileViTv3, SK attention and WIoU modules on improving the performance of the model. MSW-YOLOv8n-Seg can accurately segment cherry tomato with different maturity, and it also show strong adaptability to complex field environments. The MSW-YOLOv8n-Seg model can be integrated into picking robots to achieve automatic recognition of cherry tomato maturity, improving the picking efficiency.

The MSW-YOLOv8n-Seg model has good performance on the self-built cherry tomato dataset, which can enable optimization of architectural lightweight and segmentation. The MobileViTv3 module introduced in the backbone network can reduce the model complexity through efficient feature extraction. SK attention enhances the adaptability of the model to targets of different scales. WIoU can effectively reduce the interference of background and improve the representational ability of the target detection.

Although the MSW-YOLOv8n-Seg model has achieved promising experimental results, these outcomes are currently limited to the tests conducted on our self-built cherry tomato dataset. The model has not yet been validated for other tomato varieties or more complex agricultural scenarios. In the future, we will continue to expand the dataset to enhance the model’s robustness and generalization capabilities. Additionally, we will continue to optimize methods that how to improve the robustness of the model in challenging environmental conditions such as extreme lighting and severe occlusion and explore its integration with other emerging technologies, for instance, combining it with robot technology to achieve automatic picking.

## 5 Conclusion

To address the accuracy and efficiency problems faced by existing maturity segmentation methods when processing cherry tomatoes in complex field environments, this paper proposes a lightweight cherry tomato maturity segmentation model MSW-YOLOv8n-Seg. In order to achieve a lightweight model, we proposed the MobileViTv3 module to replace the C2f module in the original network, which uses the MobileViTv3 structure to guarantee the ability of feature picking and reduce the amount of model computation. However, lightweight operations may lead to information loss. To compensate for this, we further adopted SK attention that enhances the model’s ability to express the features of cherry tomato through integrating multi-scale features and attention weighting mechanism, thereby improving the recognition accuracy of cherry tomatoes at different maturity. The experimental results on the self-built dataset show that the proposed method achieves remarkable performance improvement. The bounding box precision, recall, and mAP@0.5 reached 90.8%, 86.3%, and 83.9% respectively. The model size was 6.0 MB. The experimental results demonstrate that MSW-YOLOv8n-Seg not only maintains high segmentation accuracy but also significantly reduces both model size and calculation amount, effectively meeting the requirements for real-time maturity detection of cherry tomatoes while indicating substantial potential for practical agricultural applications. In the future, we will combine the results of this paper with depth camera and robotic arms for in-depth research. Based on depth camera, 3D information positioning and grasping posture estimation can be achieved. Based on robotic arm, forward and inverse kinematics of robots can be solved, and the grasping way of end effectors can be studied. At the same time, to improve the application capability of the model, we will deploy it on edge devices (such as Jetson, raspberry Pi) and adopt techniques such as quantization, pruning, and distillation to accelerate the inference process.

## Data Availability

The original contributions presented in the study are included in the article/**Supplementary Material**. Further inquiries can be directed to the corresponding author.
