# Supplementary material for: Lightweight MSW-YOLOv8n-Seg: the instance segmentation of maturity on cherry tomato with improved YOLOv8n-Seg
Source: Front Plant Sci. 2026 Jan 8;16:1731580. doi: 10.3389/fpls.2025.1731580 (PMC12823800; doi:10.3389/fpls.2025.1731580)
Supplement: Supplementary file 1 [file DataSheet1.docx]

Supplementary Material

# Supplementary Figures and Tables

## Supplementary Figures


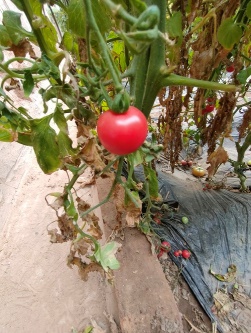

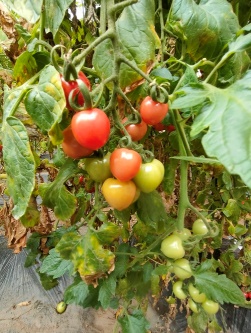

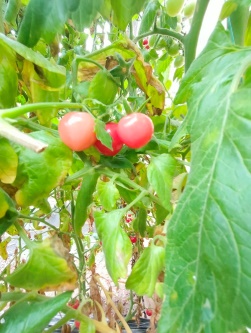

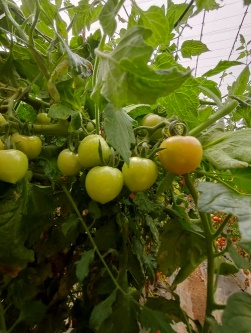


(a) (b) (c) (d)

**Supplementary Figure 1.** Sample images of cherry tomato. (a) Single-object + without branches and leaves obscured. (b) Multi-object + with fruits obscured + with branches and leaves obscured. (c) Fair-light + with branches and leaves obscured. (d) Back-light + with fruits obscured.


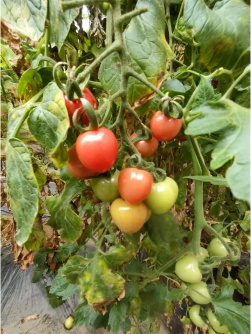

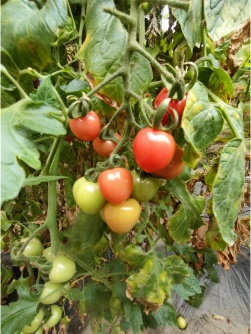

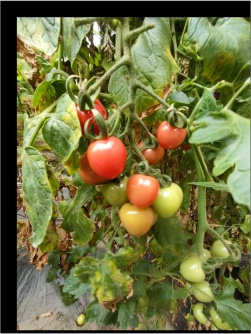

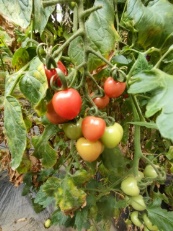


(a) (b) (c) (d)

**Supplementary Figure 2.** Data augmentation images of cherry tomato. (a) Original image. (b) Horizontal flipping image. (c) Random translation image. (d) Gaussian blur image.

**Supplementary Figure 3.** Structure of the YOLOv8-Seg network.

**Supplementary Figure 4.** Structure of the MSW-YOLOv8n-Seg network.


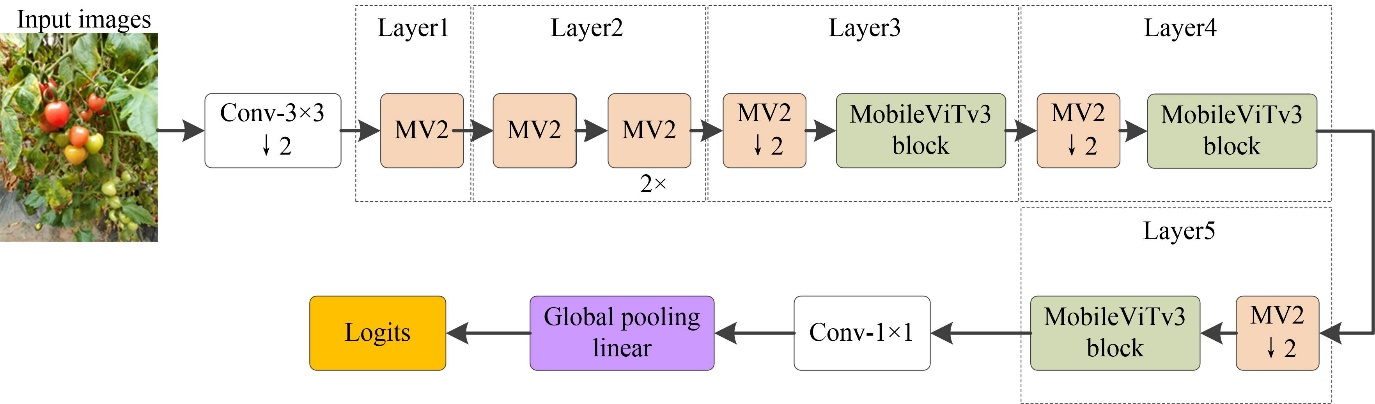


**Supplementary Figure 5.** Structure of MobileViTv3 module.


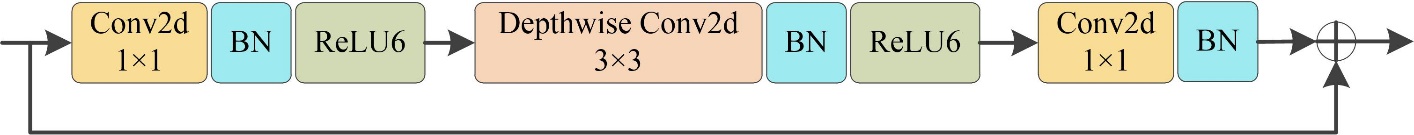


**Supplementary Figure 6.** Structure of MV2 module.


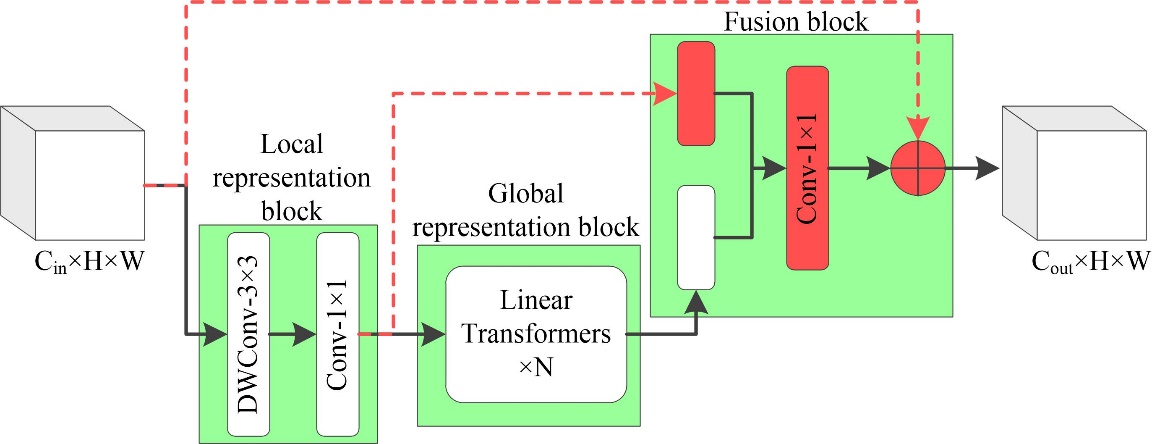


**Supplementary Figure 7.** Structure of MobileViTv3 block (MV3B).


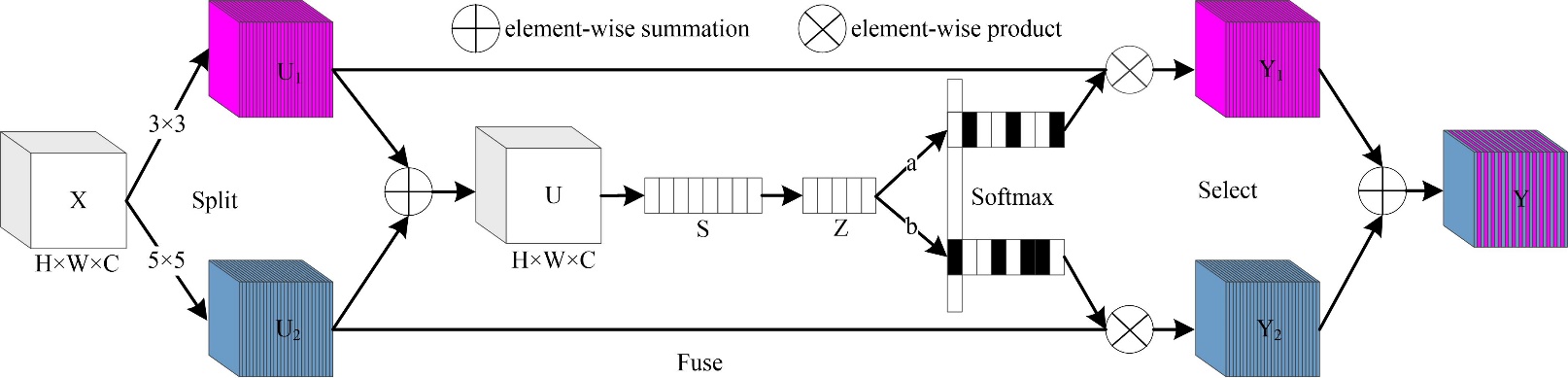


**Supplementary Figure 8.** Structure of SK attention module.


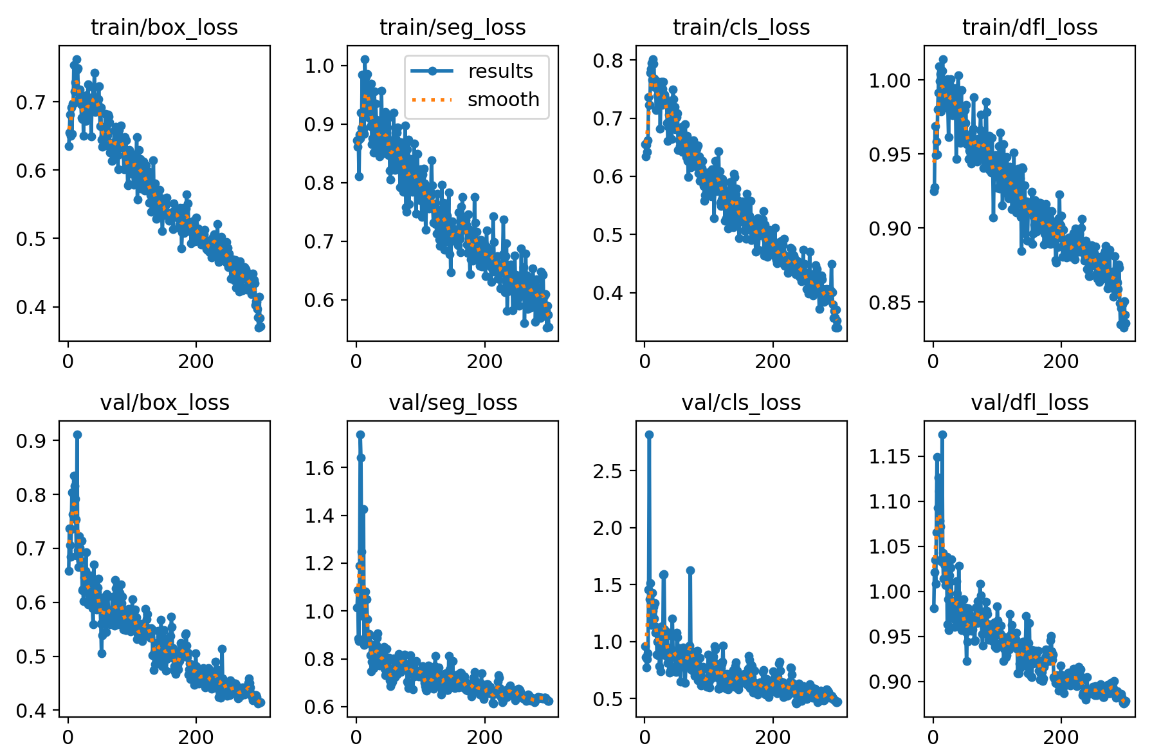


**Supplementary Figure 9.** Loss curves of MSW-YOLOv8n-Seg model on the training sets.


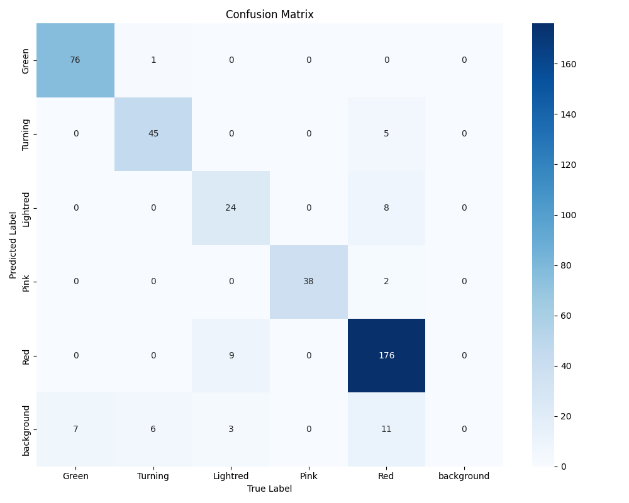

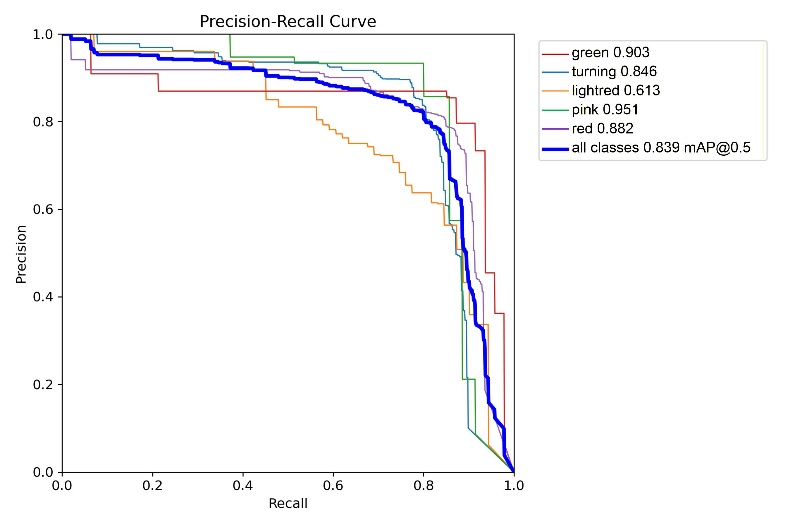


(a) (b)

**Supplementary Figure 10.** Confusion matrix and PR curve of MSW-YOLOv8n-Seg model on the test sets. (a) Confusion matrix. (b) Box PR curve.


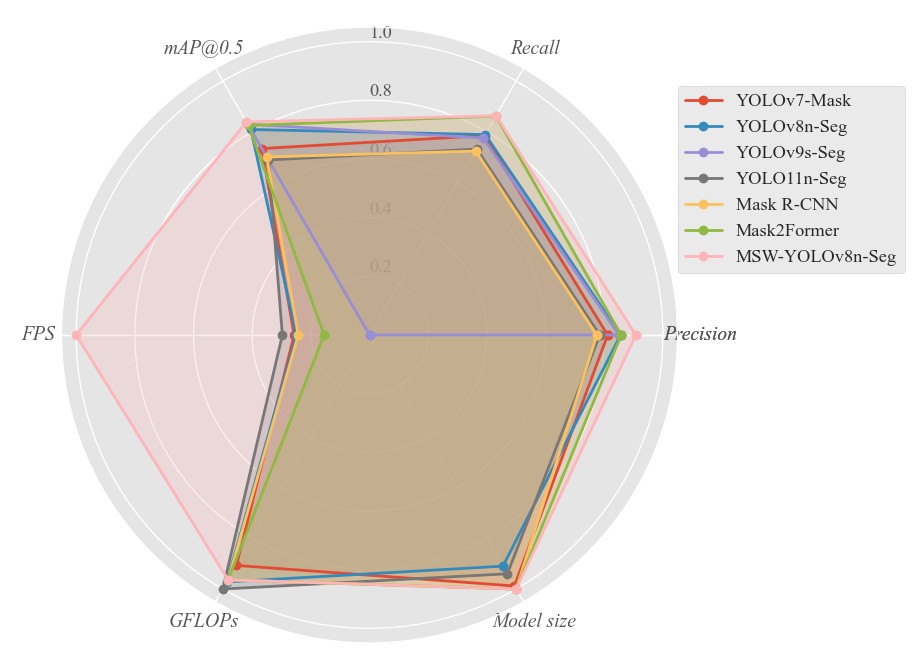


**Supplementary Figure 11.** Comparison performance of different segmentation models on six evaluation indicators.

| **Model** | Image1 | Image2 | Image3 | Image4 | Image5 |
| --- | --- | --- | --- | --- | --- |
| **Origin** | 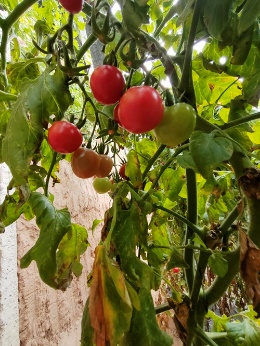 | 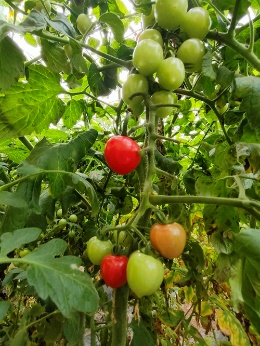 | 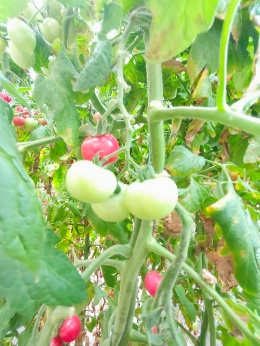 | 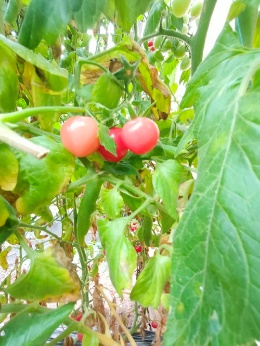 | 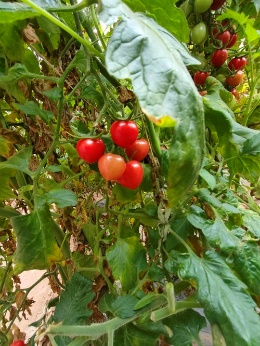 |
| **YOLOv7-Mask** | 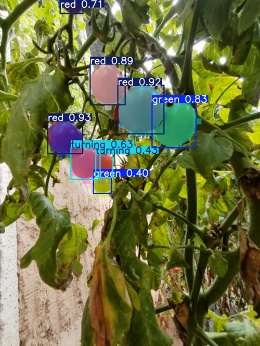 | 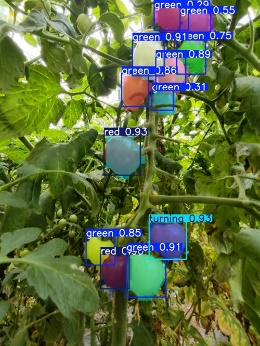 | 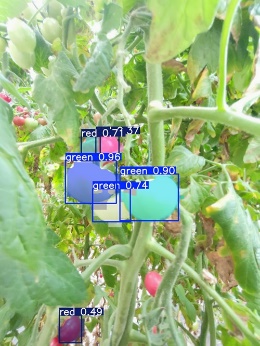 | 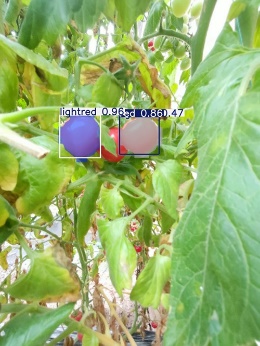 | 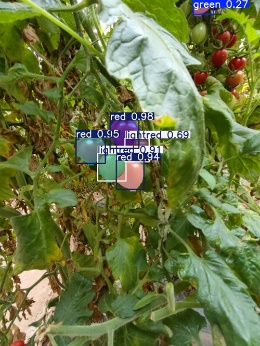 |
| **YOLOv8n-Seg** | 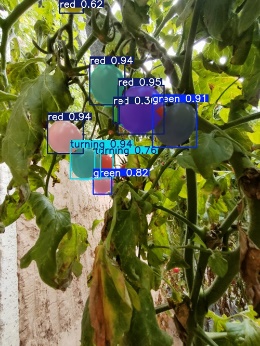 | 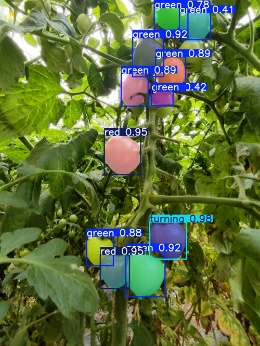 | 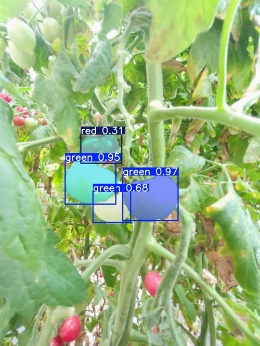 | 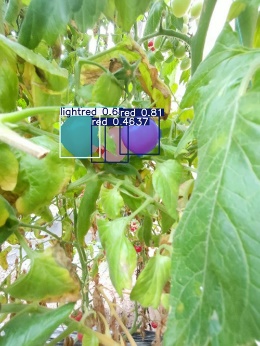 | 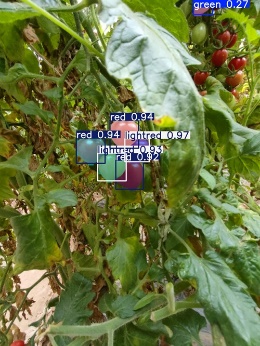 |
| **YOLOv9s-Seg** | 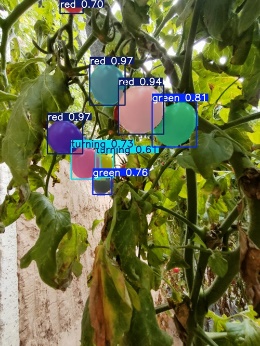 | 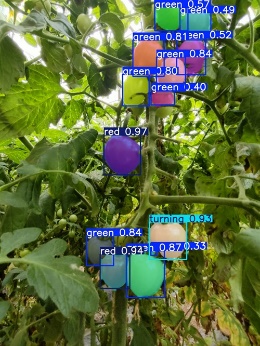 | 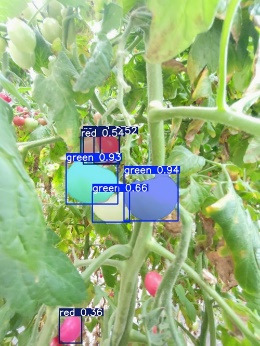 | 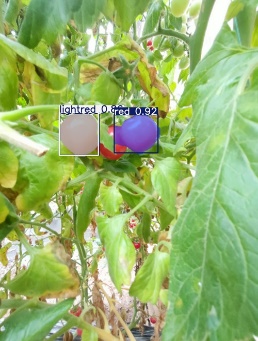 | 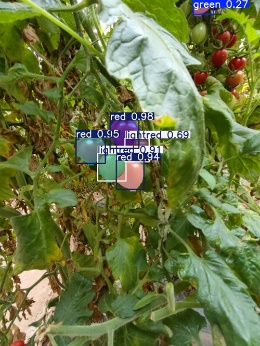 |
| **YOLO11n-Seg** | 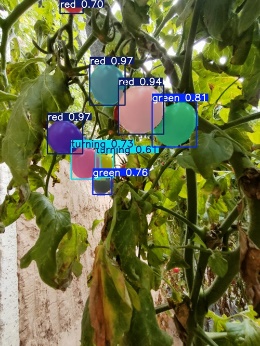 | 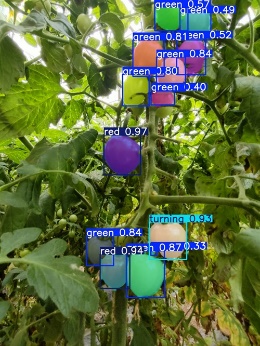 | 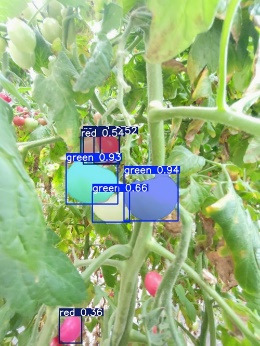 | 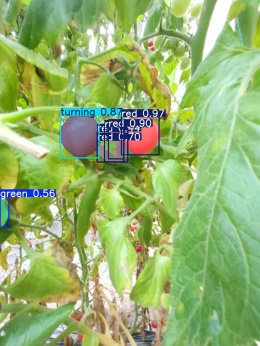 | 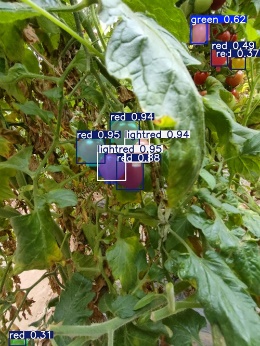 |
| **Mask R-CNN** | 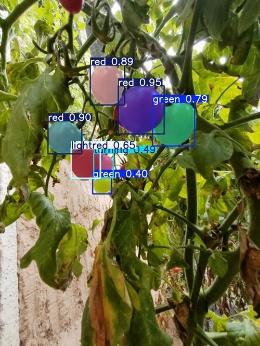 | 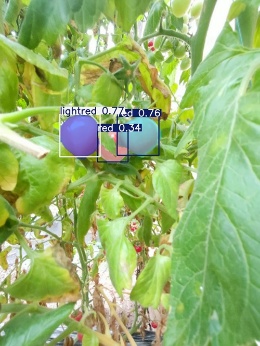 | 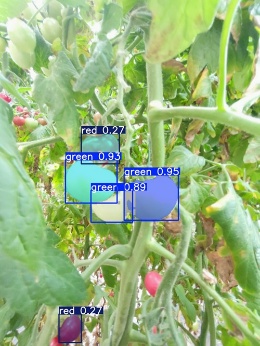 | 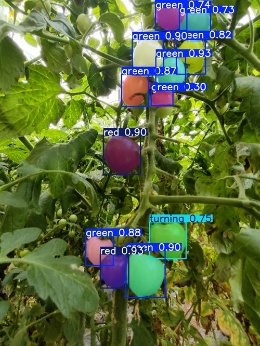 | 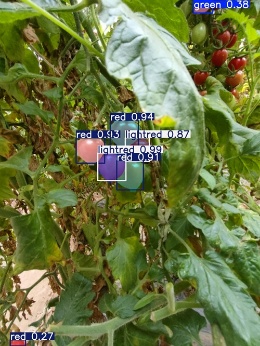 |
| **Mask2Former** | 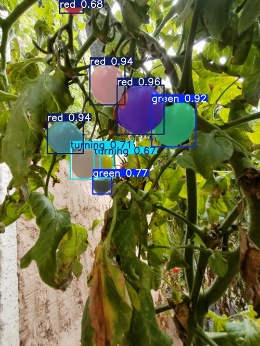 | 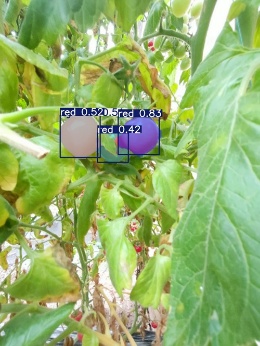 | 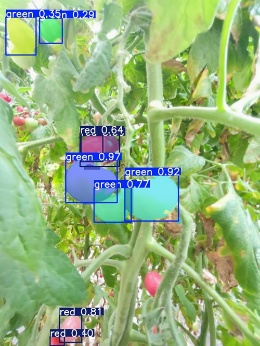 | 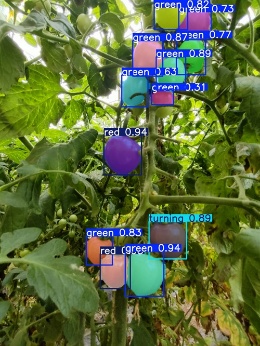 | 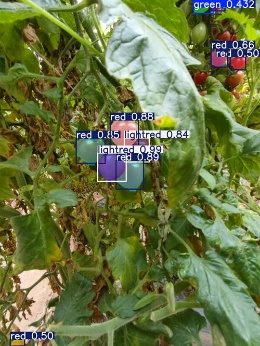 |
| **MSW-YOLOv8n-Seg** | 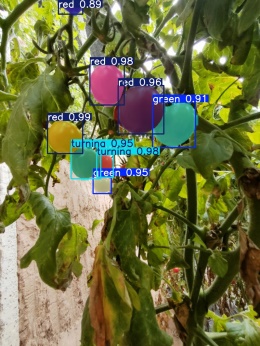 | 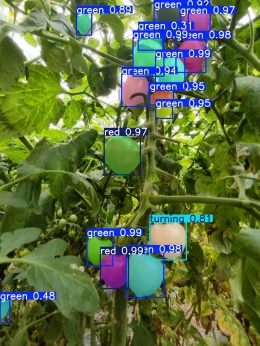 | 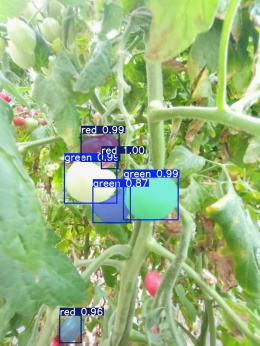 | 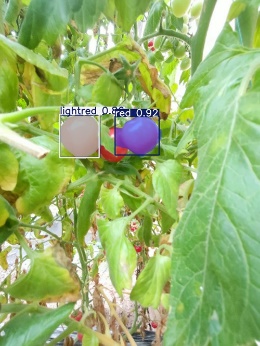 | 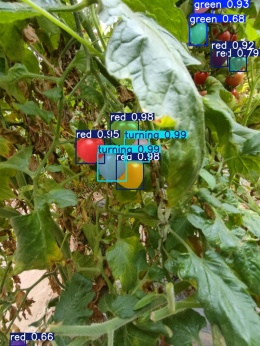 |

**Supplementary Figure 12.** Experimental results with different instance segmentation models.


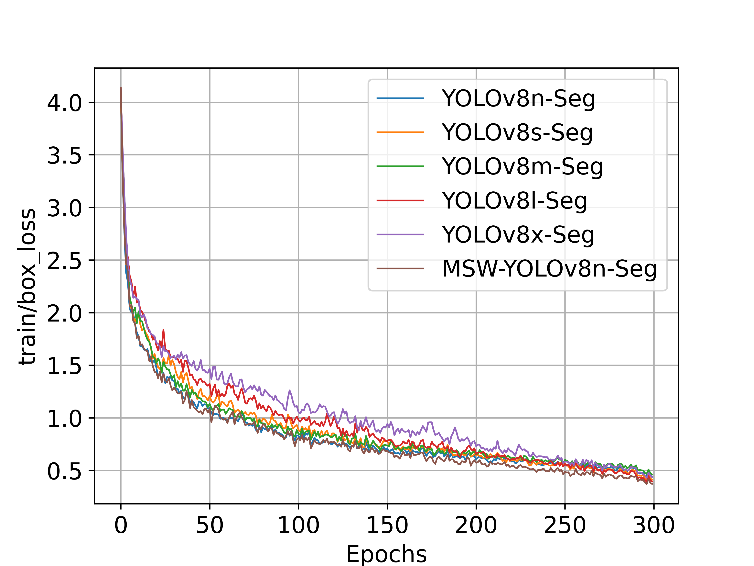

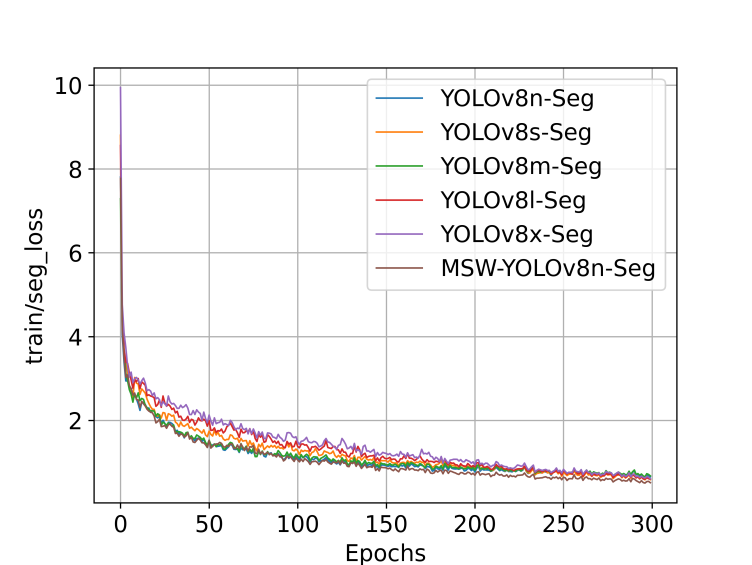

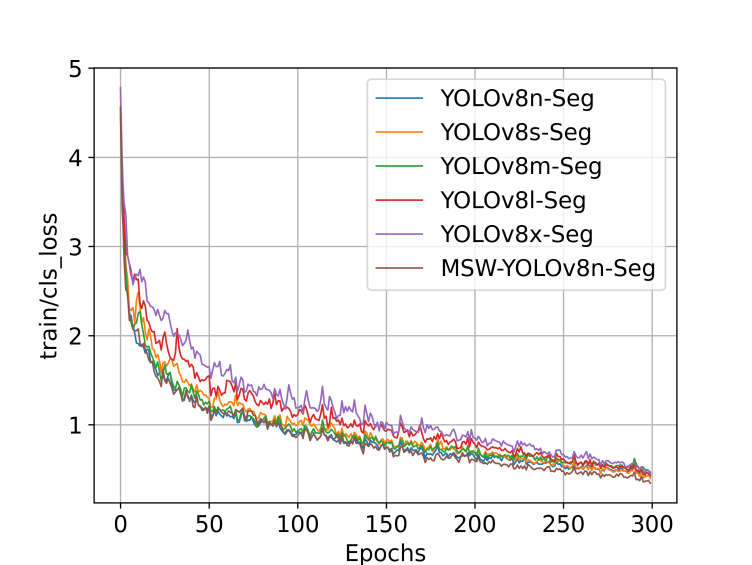

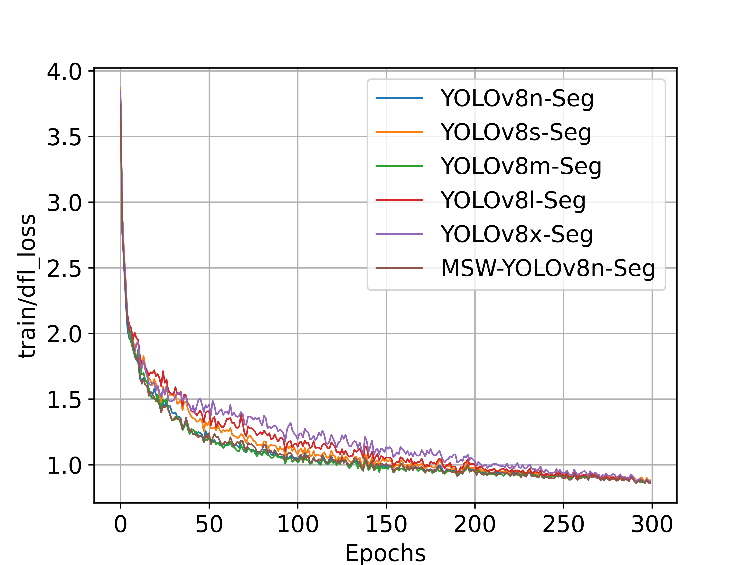


**Supplementary Figure 13.** Loss curves of different YOLOv8-Seg size models on the training sets.


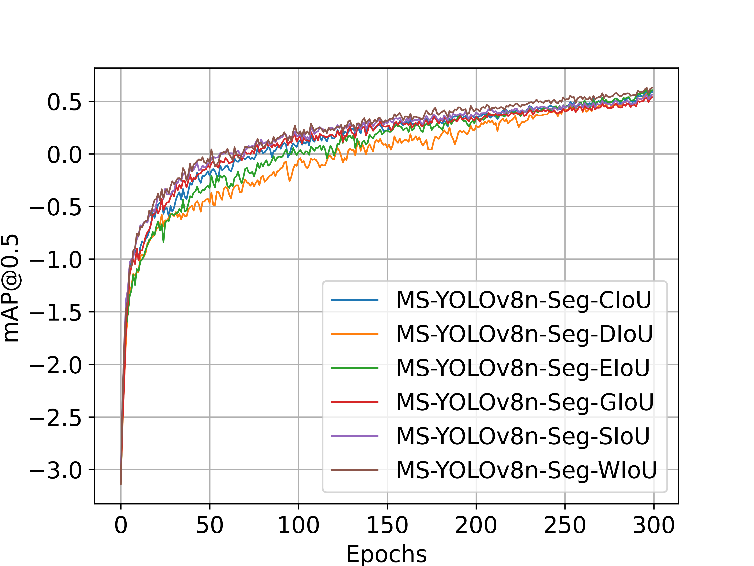

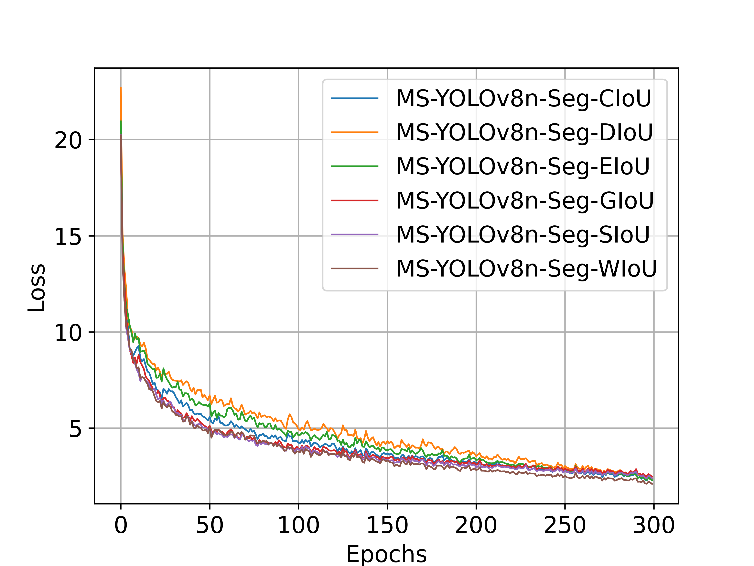


**Supplementary Figure 14.** Loss and mAP@0.5 curves corresponding to different loss functions.

## Supplementary Tables

| **Level** | **Maturity** | **Description** | **Picking situation** |
| --- | --- | --- | --- |
| 1 | Immature | The fruits have not fully grown and shaped, with green peel and no luster. | Difficult to accelerate ripening, not suitable for harvest and storage. |
| 2 | Green | The fruit have shaped and shiny, with a color change from green to white green. | Can be artificially ripened, suitable for harvest and storage, or transported over long distances. |
| 3 | Turning | Yellow or light red spots begin to appear around the navel of the frits, and the red area is less than 10%. | Suitable for harvest and storage, or transported over short distances. |
| 4 | Pre red ripe | The red area is 10%-30%. | Can be picked and fresh sales after 2 days. |
| 5 | Mid red ripe | The red area is 30%-60%. | Can be picked and fresh sales after 1 day. |
| 6 | Late red ripe | The red area is 60%-100%. | Can be picked and fresh sales the same day. |
| 7 | Over ripe | The fruit is too ripe, the flesh tissue begins to soften, the sugar content is high, and the taste is bad. | Can be used as raw materials for processing |

**Supplementary Table 1.** Maturity description of tomato in 7-levels.

| **Level** | **Maturity** | **Image** | **Description** | **Picking situation** |
| --- | --- | --- | --- | --- |
| 1 | Green | 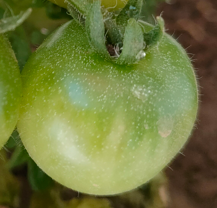 | The fruit have shaped and shiny, with a color change from green to white green. | Can be artificially ripened, suitable for harvest and storage, or transported over long distances. |
| 2 | Turning | **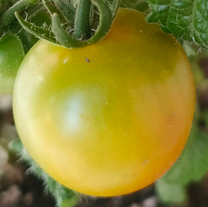** | Yellow or light red spots begin to appear around the navel of the frits, and the red area is 10%-30%. | Suitable for harvest and storage, or transported over short distances. |
| 3 | Lightred | **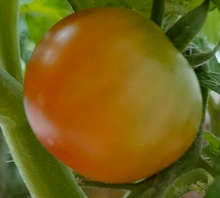** | The red area is 30%-60%. | Can be picked and fresh sales after 2 days. |
| 4 | Pink | **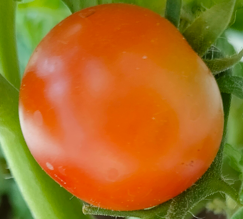** | The red area is 60%-90%. | Can be picked and fresh sales after 1 day. |
| 5 | Red | **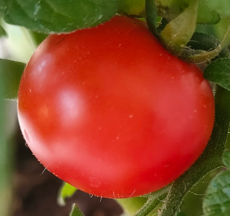** | The red area is over 90%. | Can be picked and fresh sales the same day. |

**Supplementary Table 2.** Maturity description of cherry tomato in 5-levels.

| **Maturity** | **Training set** | **Validation set** | **Test set** | **Total** |
| --- | --- | --- | --- | --- |
| Green | 347 | 26 | 83 | 456 |
| Turning | 142 | 20 | 52 | 214 |
| Lightred | 70 | 18 | 36 | 124 |
| Pink | 94 | 16 | 38 | 148 |
| Red | 624 | 56 | 202 | 882 |
| Total | 1277 | 136 | 411 | 1824 |

**Supplementary Table 3.** Annotation results of cherry tomato in different maturities.

| **No** | **Box** | | | | **Mask** | | | |
| --- | --- | --- | --- | --- | --- | --- | --- | --- |
|  | **Precision (%)** | **Recall (%)** | **mAP@0.5 (%)** | **mAP@0.5:0.95 (%)** | **Precision (%)** | **Recall (%)** | **mAP@0.5 (%)** | **mAP@0.5:0.95 (%)** |
| **1** | 89.5 | 86.1 | 83.7 | 80.2 | 89.5 | 86.1 | 83.7 | 79.8 |
| **2** | 91.2 | 87.4 | 84.0 | 78.9 | 91.2 | 87.4 | 84.0 | 77.3 |
| **3** | 90.0 | 87.1 | 83.3 | 79.5 | 90.0 | 87.1 | 83.3 | 78.5 |
| **4** | 88.9 | 85.8 | 82.5 | 78.2 | 88.9 | 85.8 | 82.5 | 77.7 |
| **5** | 91.7 | 87.9 | 84.6 | 79.1 | 91.7 | 87.9 | 84.6 | 78.2 |
| **Mean±standard deviation** | 90.3±1.0 | 86.9±0.8 | 83.6±0.7 | 79.2±0.7 | 90.3±1.0 | 86.9±0.8 | 83.6±0.7 | 78.3±0.9 |

**Supplementary Table 4.** Cross-validation results of MSW-YOLOv8n-Seg model on the validation sets.

| **Class** | **Instances** | **Box** | | | | **Mask** | | | |
| --- | --- | --- | --- | --- | --- | --- | --- | --- | --- |
|  |  | **Precision (%)** | **Recall (%)** | **mAP@0.5 (%)** | **mAP@0.5:0.95 (%)** | **Precision (%)** | **Recall (%)** | **mAP@0.5 (%)** | **mAP@0.5:0.95 (%)** |
| **Green** | 83 | 98.6 | 92.2 | 90.3 | 82.6 | 98.6 | 92.2 | 90.3 | 82.5 |
| **Turning** | 52 | 88.9 | 86.0 | 84.6 | 78.5 | 88.9 | 86.0 | 84.6 | 78.2 |
| **Lightred** | 36 | 77.0 | 67.2 | 61.3 | 60.7 | 77.0 | 67.2 | 61.3 | 60.3 |
| **Pink** | 38 | 94.4 | 99.0 | 95.1 | 93.8 | 94.4 | 99.0 | 95.1 | 93.2 |
| **Red** | 202 | 95.1 | 87.3 | 88.2 | 83.4 | 95.1 | 87.3 | 88.2 | 83.1 |
| **Total** | 411 | 90.8 | 86.3 | 83.9 | 79.8 | 90.8 | 86.3 | 83.9 | 79.5 |

**Supplementary Table 5.** Experimental results of MSW-YOLOv8n-Seg model on the test sets.

| **Complex scenarios** | **Box** | | **Mask** | |
| --- | --- | --- | --- | --- |
|  | **mAP@0.5 (%)** | **mAP@0.5:0.95 (%)** | **mAP@0.5 (%)** | **mAP@0.5:0.95 (%)** |
| **Fair-light** | 87.6 | 79.2 | 86.7 | 76.2 |
| **Back-light** | 92.3 | 84.8 | 92.3 | 83.3 |
| **Branches and leaves obscured** | 82.7 | 76.7 | 82.9 | 72.4 |
| **Multi-object with fruits obscured** | 89.7 | 80.5 | 89.0 | 78.6 |

**Supplementary Table 6.** Complex scenarios experimental results of MSW-YOLOv8n-Seg model.

| **Algorithm** | **Box** | | | | **Mask** | | | | **GFLOPs** | **Parameters** | **Model size (MB)** | **FPS(****f·s^-1^)** | **Latency(ms)** |
| --- | --- | --- | --- | --- | --- | --- | --- | --- | --- | --- | --- | --- | --- |
|  | **Precision (%)** | **Recall (%)** | **mAP@0.5 (%)** | **mAP@0.5:0.95 (%)** | **Precision (%)** | **Recall (%)** | **mAP@0.5 (%)** | **mAP@0.5:0.95 (%)** |  |  |  |  |  |
| **YOLOv7-Mask** | 81.2 | 78.5 | 73.4 | 68.7 | 81.2 | 78.5 | 73.4 | 68.7 | 16.1 | 6,213,475 | 6.5 | 15.9 | 58.9 |
| **YOLOv8n-Seg** | 85.6 | 78.9 | 80.9 | 74.4 | 85.6 | 78.9 | 81.2 | 70.5 | 12.1 | 3,210,975 | 9.8 | 15.5 | 60.6 |
| **YOLOv9s-Seg** | 85.1 | 77.5 | 83.0 | 78.5 | 85.1 | 77.5 | 83.0 | 75.7 | 71.5 | 27,839,295 | 47.9 | 3.1 | 318.2 |
| **YOLO11n-Seg** | 78.5 | 73.2 | 68.9 | 63.4 | 78.5 | 73.2 | 68.8 | 61.3 | 10.4 | 3,340,047 | 8.5 | 17.9 | 54.8 |
| **Mask R-CNN** | 77.5 | 72.4 | 70.1 | 62.5 | 77.5 | 72.4 | 70.1 | 62.5 | 358.2 | 445,473,345 | 44.0 | 15.2 | 62.8 |
| **Mask2Former** | 85.8 | 86.2 | 82.5 | 78.9 | 85.8 | 86.2 | 82.5 | 78.9 | 226.4 | 24,478,854 | 63.0 | 10.8 | 89.5 |
| **MSW-YOLOv8n-Seg (Ours)** | 90.8 | 86.3 | 83.9 | 79.8 | 90.8 | 86.3 | 83.9 | 79.5 | 12.6 | 2,661,804 | **6.0** | **52.9** | **18.2** |

**Supplementary Table 7.** Comparison results of different instance segmentation models.

| **Algorithm** | **Box** | | | | **Mask** | | | | **GFLOPs** | **Model size (MB)** |
| --- | --- | --- | --- | --- | --- | --- | --- | --- | --- | --- |
|  | **Precision (%)** | **Recall (%)** | **mAP@0.5 (%)** | **mAP@0.5:0.95 (%)** | **Precision (%)** | **Recall (%)** | **mAP@0.5 (%)** | **mAP@0.5:0.95 (%)** |  |  |
| **YOLOv8n-Seg** | 85.6 | 78.9 | 80.9 | 74.4 | 85.6 | 78.9 | 81.2 | 70.5 | 12.1 | 9.8 |
| **YOLOv8s-Seg** | 79.5 | 81.5 | 84.3 | 77.4 | 79.2 | 80.7 | 84.2 | 73.6 | 42.7 | 23.9 |
| **YOLOv8m-Seg** | 84.3 | 80.6 | 83.1 | 76.2 | 84.3 | 80.6 | 83.0 | 72.5 | 110.4 | 54.9 |
| **YOLOv8l-Seg** | 78.4 | 76.6 | 85.3 | 78.1 | 76.5 | 77.1 | 85.6 | 74.8 | 220.8 | 92.3 |
| **YOLOv8x-Seg** | 59.7 | 78.1 | 72.2 | 65.5 | 59.7 | 78.1 | 72.2 | 62.4 | 344.5 | 144.0 |
| **MSW-YOLOv8n-Seg（Our）** | 90.8 | 86.3 | 83.9 | 79.8 | 90.8 | 86.3 | 83.9 | 79.5 | 12.6 | **6.0** |

**Supplementary Table 8.** Comparison results of different YOLOv8-Seg scales.

| **Algorithm** | **Box** | | | | **Mask** | | | | **GFLOPs** | **Model size (MB)** |
| --- | --- | --- | --- | --- | --- | --- | --- | --- | --- | --- |
|  | **Precision (%)** | **Recall (%)** | **mAP@0.5 (%)** | **mAP@0.5:0.95 (%)** | **Precision (%)** | **Recall (%)** | **mAP@0.5 (%)** | **mAP@0.5:0.95 (%)** |  |  |
| **YOLOv8n-Seg** | 85.6 | 78.9 | 80.9 | 74.4 | 85.6 | 78.9 | 81.2 | 70.5 | 12.1 | 9.8 |
| **Biformer -YOLOv8n-Seg** | 71.8 | 76.3 | 83.3 | 76.4 | 81.3 | 67.5 | 82.4 | 72.3 | 12.0 | 7.7 |
| **FocalNeXt-YOLOv8n-Seg** | 80.2 | 75.4 | 83.0 | 75.5 | 80.2 | 75.4 | 83.0 | 72.3 | 11.2 | 7.2 |
| **ConvNeXtv2-YOLOv8n-Seg** | 80.6 | 79.4 | 79.0 | 71.9 | 81.1 | 78.0 | 79.1 | 70.2 | 11.4 | 6.3 |
| **Ghost Netv2-YOLOv8n-Seg** | 85.5 | 72.8 | 82.8 | 75.1 | 85.5 | 72.8 | 82.9 | 72.6 | 10.8 | 6.0 |
| **MobileViTv3-YOLOv8n-Seg** | 87.2 | 80.9 | 83.0 | 76.1 | 87.2 | 80.9 | 83.1 | 73.4 | 12.6 | 6.0 |

**Supplementary Table 9.** Comparison results of different modules in backbone.

| **Index** | **M-YOLOv8n-Seg** | **CBAM** | **EMA** | **SA** | **SimAM** | **SK** | **Box** | | | | **Mask** | | | | **Latency(ms)** |
| --- | --- | --- | --- | --- | --- | --- | --- | --- | --- | --- | --- | --- | --- | --- | --- |
|  |  |  |  |  |  |  | **Precision (%)** | **Recall (%)** | **mAP@0.5 (%)** | **mAP@0.5:0.95 (%)** | **Precision (%)** | **Recall (%)** | **mAP@0.5 (%)** | **mAP@0.5:0.95 (%)** |  |
| **1** | √ |  |  |  |  |  | 87.2 | 80.9 | 83.0 | 76.1 | 87.2 | 80.9 | 83.1 | 73.4 | 18.8 |
| **2** | √ | √ |  |  |  |  | 83.0 | 82.5 | 83.6 | 77.0 | 83.0 | 82.5 | 84.0 | 71.9 | 18.6 |
| **3** | √ |  | √ |  |  |  | 84.4 | 75.8 | 82.8 | 76.6 | 84.4 | 75.8 | 82.7 | 73.0 | 19.8 |
| **4** | √ |  |  | √ |  |  | 72.5 | 82.3 | 82.3 | 72.5 | 72.5 | 82.3 | 82.4 | 71.3 | 22.5 |
| **5** | √ |  |  |  | √ |  | 76.8 | 77.5 | 83.4 | 76.7 | 85.5 | 70.5 | 82.3 | 72.6 | 28.8 |
| **6** | √ |  |  |  |  | √ | 89.8 | 84.1 | 85.9 | 78.7 | 89.8 | 84.1 | 85.9 | 77.4 | 18.6 |

**Supplementary Table 10.** Comparison results of different attention mechanisms in neck.

| **Index** | **MS-YOLOv8n-Seg-CIoU** | **DIoU** | **EIoU** | **GIoU** | **SIoU** | **WIoU** | **Box** | | | | **Mask** | | | |
| --- | --- | --- | --- | --- | --- | --- | --- | --- | --- | --- | --- | --- | --- | --- |
|  |  |  |  |  |  |  | **Precision (%)** | **Recall (%)** | **mAP@0.5 (%)** | **mAP@0.5:0.95 (%)** | **Precision (%)** | **Recall (%)** | **mAP@0.5 (%)** | **mAP@0.5:0.95 (%)** |
| **1** | √ |  |  |  |  |  | 89.8 | 84.1 | 85.9 | 78.7 | 89.8 | 84.1 | 85.9 | 77.4 |
| **2** | √ | √ |  |  |  |  | 88.3 | 83.2 | 82.9 | 77.6 | 88.3 | 83.2 | 82.9 | 75.6 |
| **3** | √ |  | √ |  |  |  | 90.1 | 85.3 | 84.9 | 76.7 | 90.1 | 85.3 | 84.9 | 74.7 |
| **4** | √ |  |  | √ |  |  | 87.8 | 82.5 | 82.1 | 75.7 | 87.8 | 82.5 | 82.1 | 75.5 |
| **5** | √ |  |  |  | √ |  | 89.9 | 85.2 | 84.9 | 78.8 | 89.9 | 85.2 | 84.9 | 78.3 |
| **6** | √ |  |  |  |  | √ | 90.8 | 86.3 | 83.9 | 79.8 | 90.8 | 86.3 | 83.9 | 79.5 |

**Supplementary Table 11.** Comparison results of different loss functions in head.

| **Index** | **YOLOv8n-Seg** | **MobileViTv3** | **SK** | **WIoU** | **Box** | | | | **Mask** | | | |
| --- | --- | --- | --- | --- | --- | --- | --- | --- | --- | --- | --- | --- |
|  |  |  |  |  | **Precision (%)** | **Recall (%)** | **mAP@0.5 (%)** | **mAP@0.5:0.95 (%)** | **Precision (%)** | **Recall (%)** | **mAP@0.5 (%)** | **mAP@0.5:0.95 (%)** |
| **1** | √ |  |  |  | 85.6 | 78.9 | 80.9 | 74.4 | 85.6 | 78.9 | 81.2 | 70.5 |
| **2** | √ | √ |  |  | 87.2 | 80.9 | 83.0 | 76.1 | 87.2 | 80.9 | 83.1 | 73.4 |
| **3** | √ |  | √ |  | 85.2 | 82.1 | 81.5 | 75.7 | 85.2 | 82.1 | 81.5 | 74.3 |
| **4** | √ |  |  | √ | 84.7 | 79.8 | 81.0 | 75.2 | 84.7 | 79.8 | 81.0 | 74.1 |
| **5** | √ | √ | √ |  | 89.8 | 84.1 | 85.9 | 78.7 | 89.8 | 84.1 | 85.9 | 77.4 |
| **6** | √ |  | √ | √ | 84.9 | 77.8 | 79.0 | 74.2 | 84.9 | 77.8 | 79.0 | 72.8 |
| **7** | √ | √ |  | √ | 89.7 | 85.2 | 84.9 | 79.7 | 89.7 | 85.2 | 84.9 | 78.3 |
| **8** | √ | √ | √ | √ | 90.8 | 86.3 | 83.9 | 79.8 | 90.8 | 86.3 | 83.9 | 79.5 |

**Supplementary Table 12.** Comparison results of the ablation experiments.
